# Clinical and radiographic intersection of cerebral amyloid angiopathy with euglycemic diabetic ketoacidosis in the development of transient focal neurologic deficits: case report

**DOI:** 10.3389/fnimg.2025.1743623

**Published:** 2026-01-16

**Authors:** John Paul Aboubechara, Michael Saggio, Olivia Campa, Kader Karli Oguz, Ivy Nguyen

**Affiliations:** 1 Department of Neurology, University of California, Davis, Sacramento, CA, United States; 2 Department of Internal Medicine, University of California, Davis, Sacramento, CA, United States; 3 Department of Radiology, University of California, Davis, Sacramento, CA, United States

**Keywords:** case report, cerebral amyloid angiopathy, euglycemic diabetic ketoacidosis, T2 FLAIR hypointensity, transient focal neurologic episode

## Abstract

**Objectives:**

This study aimed to describe a case of transient neurologic deficits triggered by euglycemic diabetic ketoacidosis (DKA) in brain tissue at risk due to heavy cerebral amyloid angiopathy (CAA) microbleed burden, while demonstrating the rare imaging finding of reversible T2 fluid-attenuated inversion recovery (FLAIR) subcortical hypointensity.

**Methods:**

We present the clinical course, laboratory findings, and neuroimaging features of an 81-year-old man who presented with acute altered mental status and transient focal neurologic deficits.

**Results:**

The patient presented with encephalopathy, headache, left hemianopsia, left sensory neglect, and mild left upper extremity weakness. Laboratory examination showed euglycemic DKA. Brain MRI revealed findings consistent with probable CAA according to Boston Criteria 2.0, including innumerable cortical microbleeds predominantly in the right temporo-parieto-occipital lobes, with superimposed diffuse T2 FLAIR-weighted hypointensity in this region.

**Discussion:**

Reversible T2 FLAIR hypointensity has been described in hyperglycemia-associated syndromes. In this case, T2 FLAIR hypointensity likely represented metabolic dysregulation that triggered cortical dysfunction within brain regions at risk due to heavy CAA-related microbleed burden. We speculate that a common pathway for the development of the patient’s transient deficits resulted from cortical spreading depolarization (CSD), which has been associated with both CAA and hyperglycemia.

## Introduction

Cerebral amyloid angiopathy (CAA) is characterized by the deposition of amyloid-*β* protein, primarily within the walls of small-to-medium-sized cortical and leptomeningeal vessels (Greenberg and van Veluw, 2024). It is a common cause of lobar intracerebral hemorrhage in the elderly and can also manifest in patients with dementia and transient focal neurologic episodes (TFNEs) (Greenberg and van Veluw, 2024). The Boston Criteria 2.0 guides the diagnosis of CAA based on clinical symptoms and imaging features, such as lobar hemorrhage and cortical superficial siderosis (Charidimou et al., 2022; Haacke et al., 2007; Greenberg and Charidimou, 2018).

TFNEs associated with CAA have been well described and manifest as recurrent, brief, stereotyped episodes of positive (e.g., paresthesias) and negative neurological symptoms (e.g., sensorimotor deficits and vision loss) that spread contiguously over minutes to hours (Smith et al., 2021). Proposed pathophysiological mechanisms include CSD [a spreading wave of loss of ion homeostasis, change in synaptic architecture, and subsequent depression that propagates across the cortex (Kramer et al., 2016)], focal seizures, or vasoconstrictive states associated with cortical superficial siderosis or acute convexal subarachnoid hemorrhage (Smith et al., 2021).

Hyperglycemia, particularly non-ketotic hyperglycemia, has previously been associated with a variety of transient neurologic deficits, including encephalopathy, hemianopia, and other acute stroke-like syndromes, seizure-like activity, and hemichorea (Everett et al., 2023). However, euglycemic DKA has not been previously associated with transient focal neurologic deficits. Non-ketotic hyperglycemia is defined by high blood glucose without significant ketosis or acidosis, while euglycemic DKA is characterized by metabolic acidosis and ketosis in the setting of near-normal blood glucose levels. These metabolic differences may present distinct challenges to brain metabolism and function. Imaging findings associated with non-ketotic hyperglycemia include T2 FLAIR hypointensity (Paoletti et al., 2018; Raghavendra et al., 2007). T2 FLAIR hypointensity had only once previously been associated with CAA (Renard et al., 2021), but it has been observed in other conditions, such as hyperglycemia-associated seizures, ischemia, multiple sclerosis, leptomeningeal metastasis, and meningitis/meningoencephalitis (Paoletti et al., 2018; Raghavendra et al., 2007; Renard et al., 2016; De Martino et al., 2020).

In this study, we describe the case of a patient presenting with transient focal neurologic deficits in the setting of euglycemic DKA and probable CAA, with focal T2 FLAIR hypointensity overlapping regions with the heaviest burden of cortical microbleeds. We speculate that metabolic disruption wrought by hyperglycemia and acidosis may facilitate a TFNE in a patient with CAA, potentially through the induction of CSD.

## Methods

### Standard protocol approvals, registrations, and patient consents

This case report was conducted in accordance with ethical standards. Written informed consent was obtained from the patient in this case report. The study is exempt from formal ethical committee approval due to its nature as a case report involving de-identified data. Authorization for the disclosure of any potentially identifiable information, including neuroimaging data, was obtained from the patient (or their guardian) for potential publication in the journal, for use in derivative studies by the American Academy of Neurology (AAN), or for posting on the journal’s website, as applicable.

### Data access statement

As the Principal Author, I confirm that I have full access to all data used in this case report, including the neuroimaging data from the single patient. I take full responsibility for the data, its analysis, interpretation, and the conduct of the research. I have the right to publish all data, independent of any sponsor.

## Case description

**Figure 1:**
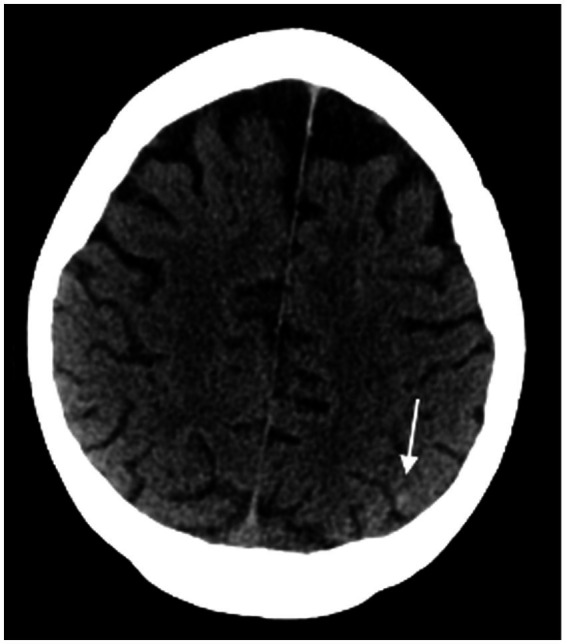
Computed tomography axial section of the head demonstrates a subtle punctate hyperdense focus in the left parietal cortex without any other observable lesions, suggesting a punctate intracerebral hemorrhage.

An 81-year-old man with hypertension, coronary artery disease, and type 2 diabetes presented with acute confusion and headache. He was found disoriented and incoherent at home. He had a year-long history of cognitive decline along with forgetfulness. Medications included aspirin, empagliflozin, and metformin. On physical examination, he was afebrile, with a blood pressure of 159/109 mmHg. Neurologic examination demonstrated disorientation, dense left homonymous hemianopsia, left-sided sensory neglect, and mild left arm weakness. Initial laboratory results included hyperglycemia (318 mg/dL), with a normal anion gap of 12, hemoglobin A1c of 11.9%, and urinalysis showed glycosuria (>1,000 mg/dL) and ketonuria. Serum glucose level improved to 175 mg/dL within 6 h without intervention. Laboratory studies, including serum chemistries, complete blood count, C-reactive protein, urine drug screen, HIV, syphilis, vitamin B1, vitamin B12, and folate, were within normal limits. A computed tomography (CT) scan of the head without contrast demonstrated punctate hyperdensity in the left parietal cortex (Figure 1). CT angiography of the head and neck was unremarkable.

The patient developed a fever of 101.5 F and thus was started on empiric encephalitis coverage. Chest radiography, urinalysis, and blood cultures were negative for other infectious sources. On hospital day 2, he developed euglycemic DKA (glucose 101 mg/dL, high anion gap of 19, and beta-hydroxybutyrate elevated at 7.54 mmol/L), which was promptly treated with intravenous hydration and insulin. Lumbar puncture and MRI were delayed due to patient agitation but were completed on hospital day 3 with general sedation. CSF analysis demonstrated a normal opening pressure of 13 cm H_2_O, a nucleated cell count of 1 cell/mm^3^, a glucose level of 112 mg/dL, and a protein level of 63 mg/dL. Further CSF testing included negative meningitis and encephalitis polymerase chain reaction panel, oligoclonal bands, and CSF cultures; thus, antibiotic treatment was discontinued.

**Figure 2:**
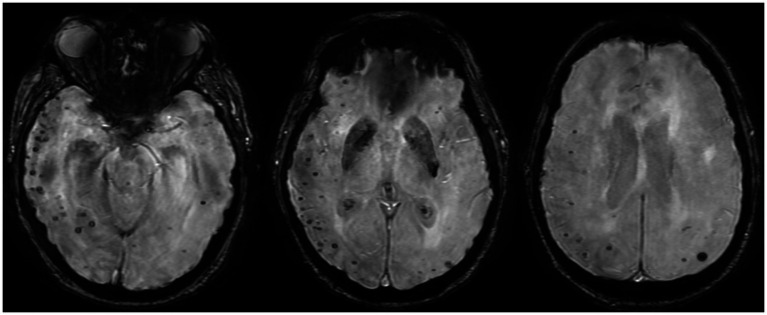
Susceptibility-weighted magnetic resonance imaging with three axial cross-sections demonstrates numerous bihemispheric punctate foci of susceptibility, especially in the right temporo-parieto-occipital lobes, suggesting microbleed.

**Figure 3:**
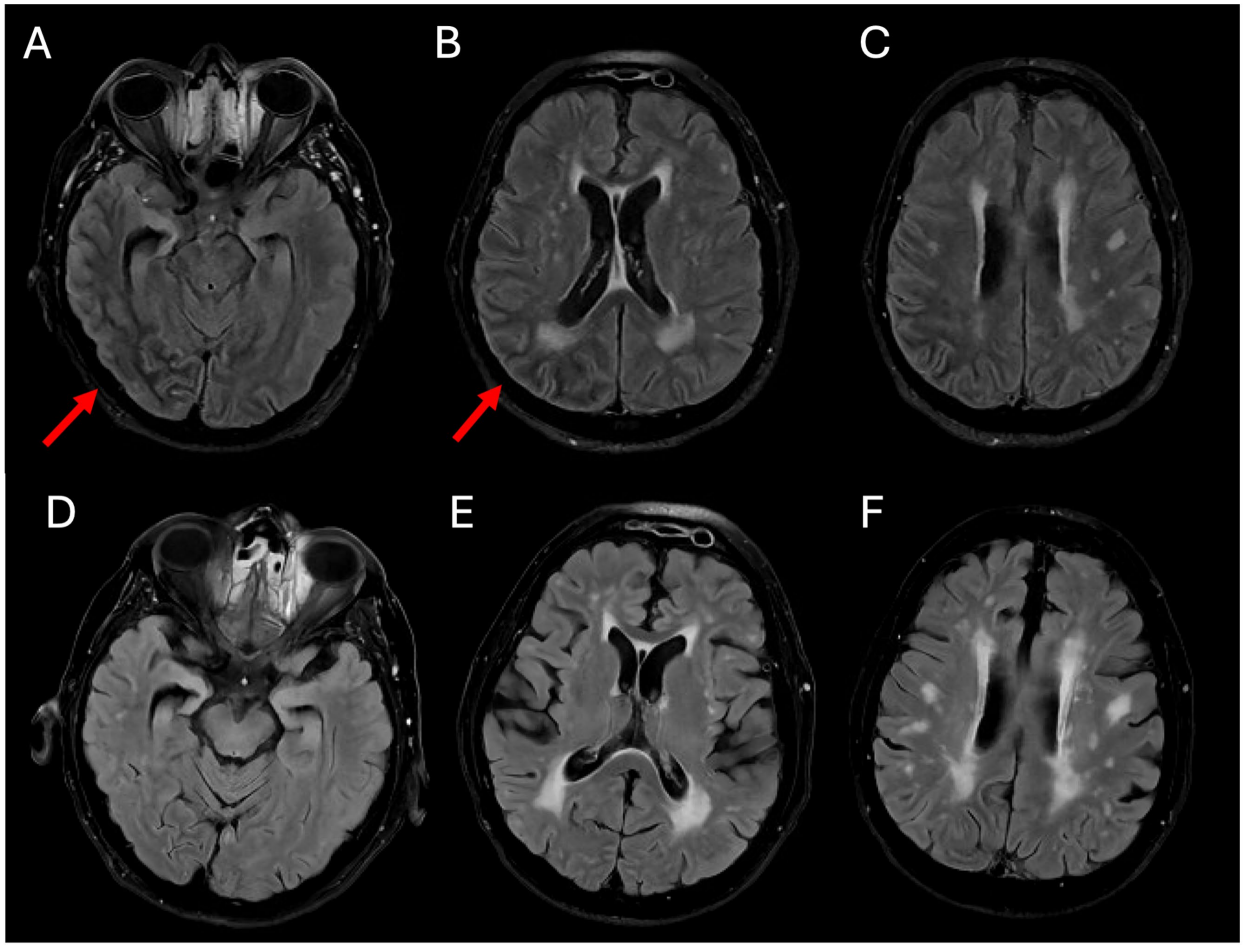
MRI brain on hospital day 3, T2-weighted fluid attenuated inversion recovery (FLAIR) images demonstrated hypointensity in the subcortical right temporal, parietal, and occipital lobes **(A–C)**. MRI brain 3 months after the onset of symptoms showed complete resolution of the previous subcortical hypointensity **(D–F)**. There are additional extensive chronic white matter lesions of microangiopathy presenting as partly confluencing scattered hyperintense lesions on T2 FLAIR imaging **(D–F)**.

**Figure 4:**
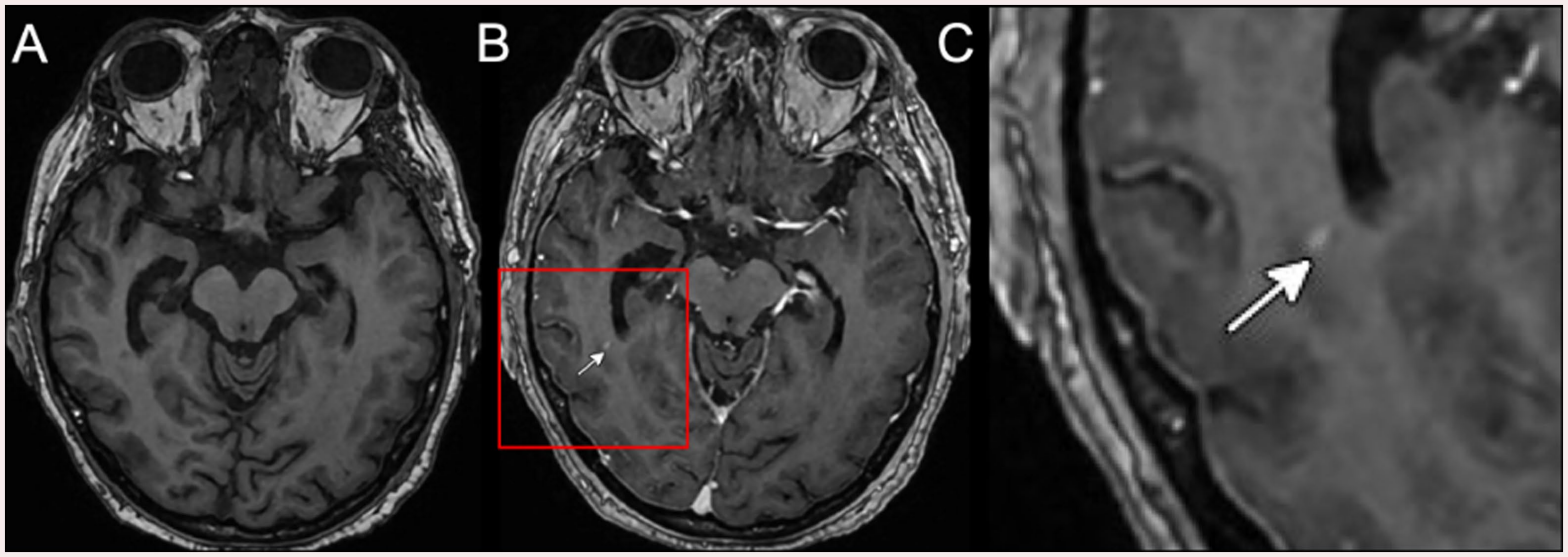
MRI brain on hospital day 3, demonstrating a single punctate focus of contrast enhancement in the right medial temporal lobe. **(A)** T1 pre-contrast sequence, **(B)** T1 post-contrast sequence, and **(C)** magnified image of the T1 post-contrast image region of interest shown in panel **(B)**.

Brain MRI with and without contrast revealed numerous abnormal findings as follows: (i) numerous cortically based punctate foci of susceptibility, predominantly in the right than the left hemisphere, with 291 in the right hemisphere and 76 in the left hemisphere (ratio of right/left of 3.8) (Figure 2), one of which coincides with the punctate hyperdensity observed on the prior CT scan (Figure 1); (ii) diffuse T2 FLAIR hypointensity throughout the white matter of the right temporal, parietal, and occipital lobes (Figures 3A–C); and (iii) a single punctate focus of contrast enhancement in the right medial temporal lobe within the region of highest cortical microbleed burden but that did not co-localize with any susceptibility signal (Figures 4A–C). Continuous electroencephalogram over 24 h demonstrated focal right posterior slowing, but there was no evidence of epileptiform activity. By hospital day 3, the patient’s encephalopathy had improved, and his left-sided focal neurologic deficits had resolved.

His history of cognitive decline and MRI findings of numerous cortical microbleeds met criteria for CAA (Charidimou et al., 2022). As such, he was suspected of having experienced a prolonged TFNE in the setting of probable CAA and euglycemic DKA. The patient was discharged home on hospital day 5 at his baseline cognitive and neurologic status. Aspirin was discontinued due to the increased risk of hemorrhage in CAA (Biffi et al., 2010). Euglycemic DKA was attributed to the SGLT2 inhibitor empagliflozin, which was discontinued, and his diabetes regimen was optimized. Repeat brain MRI at 3-month follow-up demonstrated interval resolution of the subcortical T2 FLAIR hypointensity (Figures 3D–F). Six months after discharge, he underwent outpatient cognitive screening with a Montreal Cognitive Assessment (MOCA) score of 17/30. Screening laboratory studies for reversible neurocognitive conditions demonstrated a normal Vitamin B12 level, normal thyroid-stimulating hormone level, non-reactive human immunodeficiency virus, and non-reactive syphilis. Subsequent neuropsychological testing was performed, which demonstrated frontal-subcortical dysfunction with executive dysfunction also impacting learning/memory. He was given a diagnosis of mild cognitive impairment, bordering on major neurocognitive disorder. Etiology was believed to be related to CAA and moderate cerebrovascular white matter disease, with contributing factors including obstructive sleep apnea, insomnia, and chronic pain. Alzheimer’s disease was considered less likely given executive-predominant findings and the absence of amnestic quality. At the 6-month and 12-month follow-up visits in the Neurology clinic, the patient had remained clinically stable without any recurrent episodes of transient neurologic deficits (Table 1).

**Table 1:** Timeline of clinical events.

Timepoint	Clinical event and status	Relevant data and imaging	Management and diagnosis
Past medical history	1-year history of cognitive decline. Comorbidities: HTN, CAD, and T2DM.	Baseline HbA1c: 11.9%	Home Meds: Empagliflozin, Metformin, and Aspirin.
Admission (day 1)	Acute onset of confusion and headache. Exam: Disoriented, left homonymous hemianopsia, left sensory neglect, and mild left arm weakness.	Labs: Glucose: 318 mg/dL, anion gap: 12 (normal). UA: >1,000 mg/dL glucose, +ketones. CT Head: Punctate hyperdensity (left parietal).	Admitted for workup. Started on empiric antibiotics/antivirals for fever (101.5 °F).
Hospital day 2	Development of metabolic acidosis.	Labs: Glucose: 101 mg/dL (Euglycemic); anion gap: 19 (High); and beta-hydroxybutyrate: 7.54 mmol/L.	Diagnosis: Euglycemic DKA. Action: Empagliflozin stopped. IV fluids and insulin protocol initiated.
Hospital day 3	Resolution of encephalopathy and focal deficits (hemianopsia/neglect).	MRI Brain: >290 microbleeds (bi-hemispheric), T2 FLAIR hypointensity (right temporo-parieto-occipital), and single focus of contrast enhancement. CSF: 1 nucleated cell per cubic millimeter, glucose 112 mg/dL, and protein of 63 mg/dL. EEG: Right posterior slowing.	Antibiotics discontinued. Aspirin discontinued (CAA diagnosis).
Hospital day 5	Patient at neurologic baseline.	N/A	Discharged home.
3 months post-discharge	Outpatient follow-up.	MRI brain: Complete resolution of T2 FLAIR hypointensity. Chronic microbleeds and white matter changes persist.	Continued supportive care.
6-month post-discharge	Cognitive evaluation.	Cognitive testing: MoCA 17/30. Neuropsychology: frontal-subcortical dysfunction.	Diagnosis of mild cognitive impairment (likely vascular/CAA etiology).

## Discussion

The case illustrates a prolonged TFNE in the setting of newly diagnosed probable CAA and acute non-ketotic hyperglycemia that evolved into euglycemic DKA with empagliflozin usage. The patient’s TFNE in the setting of chronic, progressive cognitive impairment and numerous cortical microbleeds on neuroimaging strongly supports the diagnosis of probable CAA as per Boston Criteria 2.0 (Charidimou et al., 2022). The episode of left-sided hemianopsia and neglect to resolve within 48 h may be due to hyperglycemia-related hemianopia or atypical prolonged CAA-related TFNE. While hyperglycemic hemianopia, typically associated with focal seizures and reversible T2 FLAIR subcortical hypointensity, is a recognized complication of non-ketotic hyperglycemia (De Martino et al., 2020), it has not been reported in the context of euglycemic DKA or CAA-related cortical microbleeds. A focal seizure with post-ictal focal deficits is possible; however, no ictal or inter-ictal activity was observed, and the duration of deficits was greater than that typically noted for post-ictal paresis (Gallmetzer et al., 2004). We speculate that the prolonged duration of the TFNE in this case could be explained by the concurrent and prolonged period of euglycemic DKA with SGLT-2 use. SGLT-2 inhibitors, such as empagliflozin, lower the threshold for glucosuria, maintaining near-normal serum glucose even during states of insulin deficiency and ketosis (Chow et al., 2023). This ‘euglycemic’ state can delay diagnosis and prolong the duration of acidosis (Chow et al., 2023). We speculate that this prolonged metabolic acidosis contributed to the extended duration of the TFNE in this case.

The imaging novelty in this case was the presence of reversible right posterior T2 FLAIR subcortical hypointensity localized to regions of densest cortical microbleeds and corresponding well with the patient’s left visual field cut and neglect, suggesting that this signal change was a marker of the underlying acute pathophysiologic process. Though uncommon, subcortical T2 FLAIR hypointensity has been noted in other conditions (Paoletti et al., 2018; Raghavendra et al., 2007; Renard et al., 2016; De Martino et al., 2020; Lee et al., 2002). The mechanism is unclear, but it has been suggested to represent cytotoxic edema, the accumulation of paramagnetic substances, or altered water content (Lee et al., 2002). This rare finding has been observed once previously in the setting of CAA-related TFNE (Renard et al., 2021). However, the case represented by Renard et al. occurred in the context of superficial siderosis, whereas our patient presented with predominant cortical microbleeds and euglycemic DKA, suggesting that T2 FLAIR hypointensity may be a shared biomarker for metabolic stress in amyloid-laden vessels, regardless of the specific trigger.

Finally, we observed a single punctate focus on contrast enhancement. While often associated with inflammatory CAA, contrast extravasation has recently been described in CAA-related TFNEs as a marker of acute blood–brain barrier disruption (van den Brink et al., 2025). Notably, in our case, this enhancement did not co-localize with a microbleed (susceptibility signal), suggesting that metabolic stress can disrupt the blood–brain barrier in susceptible tissue independently of frank vessel rupture.

The patient’s presentation likely resulted from an interaction between both CAA and transient hyperglycemia that evolved into euglycemic DKA. Three potential interactions can be posited in this case. First, DKA could be the primary cause of the patient’s deficits, causing fluid shifts and metabolic dysfunction in brain tissue at risk due to significant CAA-related microbleed burden (Marren et al., 2022). Second, DKA could have facilitated CSD triggered by prolonged CAA-related TFNE (Smith et al., 2021). Third, metabolic dysfunction could have triggered autonomic vascular dysfunction, resulting in increased focal cerebral blood flow and a syndrome similar to posterior reversible encephalopathy syndrome. The resolution of imaging findings and symptoms following metabolic correction suggests a transient metabolic-vascular interaction in a vulnerable CAA-affected brain.

## Conclusion

We report a novel case of an episode of hemianopia and neglect triggered by the interaction between euglycemic DKA and CAA. It also depicts the striking co-localization of T2 FLAIR hypointensity with CAA-related microbleeds. We propose that metabolic disturbances such as hyperglycemia may trigger focal neurologic deficits in brain regions at risk of CAA, possibly through CSD. Further investigation is needed to understand the significance and pathophysiology of T2 FLAIR hypointensity and its implications for CAA-related events.

Euglycemic DKA may trigger transient focal neurologic episodes in patients with cerebral amyloid angiopathy, and T2 FLAIR hypointensity on MRI may serve as a biomarker of this pathophysiologic process.

## Data Availability

The original contributions presented in the study are included in the article/supplementary material; further inquiries can be directed to the corresponding authors.
